# A highly multiplexed broad pathogen detection assay for infectious disease diagnostics

**DOI:** 10.1371/journal.pntd.0006889

**Published:** 2018-11-05

**Authors:** Jeffrey W. Koehler, Christina E. Douglas, Timothy D. Minogue

**Affiliations:** Diagnostic Systems Division, United States Army Medical Research Institute of Infectious Diseases, Fort Detrick, Maryland, United States of America; Molecular Biology Unit (MBU), INDIA

## Abstract

Rapid pathogen identification during an acute febrile illness is a critical first step for providing appropriate clinical care and patient isolation. Primary screening using sensitive and specific assays, such as real-time PCR and ELISAs, can rapidly test for known circulating infectious diseases. If the initial testing is negative, potentially due to a lack of developed diagnostic assays or an incomplete understanding of the pathogens circulating within a geographic region, additional testing would be required including highly multiplexed assays and metagenomic next generation sequencing. To bridge the gap between rapid point of care diagnostics and sequencing, we developed a highly multiplexed assay designed to detect 164 different viruses, bacteria, and parasites using the NanoString nCounter platform. Included in this assay were high consequence pathogens such as Ebola virus, highly endemic organisms including several *Plasmodium* species, and a large number of less prevalent pathogens to ensure a broad coverage of potential human pathogens. Evaluation of this panel resulted in positive detection of 113 (encompassing 98 different human pathogen types) of the 126 organisms available to us including the medically important Ebola virus, Lassa virus, dengue virus serotypes 1–4, Chikungunya virus, yellow fever virus, and *Plasmodium falciparum*. Overall, this assay could improve infectious disease diagnostics and biosurveillance efforts as a quick, highly multiplexed, and easy to use pathogen screening tool.

## Author summary

Identifying the causative agent in an acute febrile illness can be challenging diagnostically, especially when organisms in a particular region have overlapping clinical presentation or when that pathogen’s presence is unexpected. Ebola virus, for example, was not considered in an acute febrile illness differential diagnosis in West Africa until the explosive outbreak in 2013 presented the risk of infection. Besides the cost and time of screening a single patient sample for a large number of pathogens, limited sample volumes place further restrictions on what assays can be applied. Here, we developed a broad pathogen screening assay targeting 164 different human pathogens and show positive detection of over 100 of the organisms on the panel including Ebola virus, *Plasmodium falciparum*, and a large number of rare pathogens. The hands on time and sample volume requirement is minimal. The assay performed well in mock clinical and human clinical samples, demonstrating the clinical utility of this assay in cases where the initial diagnostic testing results in negative results. Our results provide a framework for further validation studies that would be required for formal clinical diagnostic applications.

## Introduction

Appropriate diagnostic assay selection for infectious diseases depends on multiple parameters including clinical presentation and endemic pathogens known to circulate within a specific geographic region. Rapid point-of-care PCR [1, 2] and lateral flow immunoassays [3, 4] as well as more complex PCR [5–7] and laboratory based antigen capture ELISAs [8, 9] can generate a clinically actionable diagnosis in patients presenting with an acute febrile illness. These assays are sensitive, rapid, and relatively inexpensive, making this testing approach ideal for initial diagnostic testing. If these assays are negative, however, additional testing including increasingly multiplexed assays and agnostic next-generation sequencing can be utilized.

Multiplexed assays such as the MAGPIX [10, 11] or multiplexed real-time PCR [12–15] can increase the number of targets being tested. For example, Munro and colleagues described a multiplexed PCR assay with detection on the MAGPIX or Luminex instruments capable of detecting multiple influenza viruses with performance similar to real-time PCR [11]. Similarly, a multiplexed real-time RT-PCR assay, developed by Santiago and colleagues and approved by the FDA as an *in* vitro diagnostic device, detects all four dengue virus serotypes in a single tube reaction [15].

In cases where the initial testing methods do not result in positive pathogen identification, next-generation sequencing (NGS) is another alternative for clinically actionable infectious disease diagnostics [16]. However, metagenomic sequencing can be challenging due to a large host background, necessitating high sequencing depth to generate sufficient on target reads for pathogen detection. Targeted NGS, in which a specific signature is amplified [17, 18] or enriched from a complex sample using hybridization [19], can increase pathogen specific reads sufficiently to allow detection on desktop sequencers such as the Ion Torrent or the MiSeq. Using these approaches, however, adds time-to-answer due to library preparation, sequencing, and analysis.

A potential solution described here is the use of the NanoString nCounter platform for highly multiplexed pathogen detection. This system utilizes direct hybridization and detection of a nucleic acid target and can be highly multiplexed (up to 800 different targets). Since this technology has been successfully implemented for quantitative gene expression studies [20–22], we investigated whether this platform could be used for broad, targeted pathogen detection in a situation where rapid testing (ex. real-time PCR) was negative. In this context, we developed and evaluated a panel containing 195 different assay targets against 164 different viruses, bacteria and parasites. Overall, this panel was not as sensitive as real-time PCR; however, this assay successfully identified multiple pathogens quickly, demonstrating utility as a pathogen screening assay.

## Materials and methods

### Viruses, parasites, and bacteria

All organisms used in this study (listed in S1 File) are maintained at United States Army Medical Research Institute of Infectious Diseases (USAMRIID) or were provided by the Unified Culture Collection (UCC) or the American Type Culture Collection (ATCC, Manassas, VA). Samples included bacterial, parasite DNA, cell culture supernatant from virus-infected cells treated with TRIzol LS (ThermoFisher Scientific, Waltham, MA) or gamma irradiation. Total nucleic acid from each unpurified sample was extracted using the EZ1 Virus Mini Kit v2.0 (Qiagen, Valencia, CA) with the EZ1 robot (Qiagen) according to the manufacturer’s instructions. Total nucleic acid was eluted in 90 μl elution buffer.

Due to a limited supply, *Coxiella burnetii* DNA was amplified using the REPLI-g Whole Genome Amplification Kit (Qiagen) according to the manufacturer’s instructions. The number of *C*. *burnetii* genome equivalents (GE) was approximated using the genome of *C*. *burnetii* RSA493 (GenBank# NC_002971) and the C+G (42.7%) and A+T (57.3%) genome percentages. Based on these calculations, 1 GE is approximately 2.05 fg. The approximate number of GE for *Plasmodium falciparum* 3D7 DNA (ATCC) was similarly determined to be approximately 23.89 fg.

### NanoString broad pathogen panel

A custom Broad Pathogen Detection Assay (BPDA) targeting a broad panel of medically important viruses, bacteria, and parasites was designed and acquired from NanoString Technologies (Seattle, WA). Using 195 different capture and reporter probes, this assay targeted 164 different pathogens of concern for human health (S1 File). After initial testing showed lower than desired assay sensitivity, nested primers targets were designed by NanoString using Primer3 software [23–25]; see S1 File for the sequences. These multiplexed primers were used in a multiplexed target enrichment (MTE) reaction to amplify the capture/reporter target prior to detection. Primer pairs for 4 probe targets could not initially be designed and were redesigned for incorporation into a subsequent MTE iteration.

Individual MTE primer pairs for all available pathogens were evaluated for amplicon generation using SuperScript One-Step with Platinum *taq* (Thermo Fisher Scientific) with the following cycling conditions: 50°C for 15 minutes, 95°C for 5 minutes, 40 cycles of 95°C for 30 seconds, 60°C for 1 minute and 72°C for 1 minute. The final reagent concentrations per 20 μL reaction were: 1X Reaction Mix, 4 mM MgSO_4_, 0.25 mg/mL BSA, 50 μM primers, 0.4 units of Platinum *Taq*. Amplicon generation was visualized on the 2100 Bioanalyzer (Agilent Technologies, Santa Clara, CA) using the DNA 1000 Kit (Agilent Technologies).

After confirming successful amplification using selected individual primer pairs, all primers were combined into a single 500 nM primer mixture for the MTE reaction. Sample cDNA was generated by adding 4 μL purified total nucleic acid to 1μL of SuperScript VILO MasterMix (Thermo Fisher Scientific) and incubating at 25°C for 10 minutes, 42°C for 60 minutes, and 5 minutes at 85°C. The sample was then added to 5 μL of TaqMan PreAmp Master Mix (Thermo Fisher Scientific) and 1 μL of the 500 nM primer mixture. MTE used the following cycling conditions: 94°C for 10 minutes, then 18 cycles of 94°C for 15 seconds, 60°C for 4 minutes, and a 4°C hold. The entire enriched sample (11 μL) was used for detection on the NanoString nCounter platform.

Pathogen nucleic acid (total nucleic acid and MTE amplified nucleic acid) was initially denatured at 95°C for 5 minutes and immediately transferred to ice for 2 minutes. Next, 5 μl of each denatured sample or the entire MTE reaction (11 μl) was added to a 20 μl NanoString master mix containing the BPDA Reporter Codeset plus 130 μL hybridization buffer followed by 5 μl of the BPDA Capture Codeset. Reactions were immediately placed on a thermocycler for an overnight incubation at 65°C (for ~16 hours), loaded onto a sample cartridge using the nCounter Prep Station, and scanned using the NanoString nCounter Digital Analyzer. A sample was called positive for a specific target if the number of counts was greater than the average of the internal negative controls for that target plus three times the standard deviation of the negative controls.

### MTE amplification assessment

The impact of the MTE reaction on sensitivity was assessed using SYBR Green real-time PCR assays using primers internal to the MTE primers [See S1 File for Bundibugyo virus (BDBV), Marburg virus (MARV), Ebola virus (EBOV), influenza B virus, and *P*. *falciparum* assay information]. Total nucleic acid for each organism was serially diluted and amplified by MTE. The levels of enrichment were measured by real-time RT-PCR using Superscript RT-PCR reagents (Thermo Fisher Scientific) and SYBR Green. The cycling conditions for the SYBR Green RT-PCR assays were: 50°C for 15 minutes, 95°C for 5 minutes, 45 cycles of 95°C for 5 seconds and 60°C for 20 seconds, and a final melt step from 60°C—95°C at a rate of 0.2°C per second. The final reagent concentrations per 20 μL reaction were as follows: 1X Reaction Mix, 3mM MgSO_4_, 0.25 mg/mL BSA, 1 μM primers, 2X SYBR Green, and 1 unit of Platinum *Taq*.

### Assay limit of detection and reproducibility

Following optimization and characterization of the MTE reaction, assay performance was determined utilizing the MTE reaction and detection on the NanoString platform. A preliminary limit of detection (LOD) for BDBV, MARV, EBOV, influenza B virus, and *P*. *falciparum* was determined with and without MTE by serially diluting organism and testing for positive detection. The preliminary LOD was defined as the lowest concentration of organism having all three replicates testing positive. Testing at the preliminary LOD was repeated (ten replicates) without MTE for BDBV and MARV Angola and with MTE for influenza B virus, BDBV, and MARV Angola to show assay reproducibility. Similarly, LODs were conducted using existing real-time RT-PCR assays as previously described [26, 27].

### Clinical samples

Mock clinical samples were prepared in order to evaluate the ability of the BPDA to detect samples that had been extracted from whole human blood. Dengue virus serotype 3 (DENV-3) in TRIzol LS and gamma-irradiated EBOV were diluted in human whole blood treated with EDTA (Bioreclamation, Westbury, NY) in 200 μl samples. Samples were extracted using the Qiagen EZ1 XL Advanced with the EZ1 Virus Mini kit 2.0 by adding an equal volume of ATL buffer (Qiagen) to each sample prior to being placed on the automated instrument for extraction. Each sample was run in triplicate with and without MTE amplification prior to being tested with the pathogen panel.

### Ethics statement

Since the BPDA assay could be a useful diagnostic tool, de-identified human clinical samples were tested. These samples were acquired through USAMRIID’s Special Pathogens Laboratory. All samples were de-identified prior to use, and all studies were conducted in compliance with United States Department of Defense, federal, and state statutes and regulations relating to the protection of human subjects, and adheres to principles identified in the Belmont Report. All data and human subjects research were gathered and conducted for this publication under an Institutional Review Board approved determination FY17-31 as defined by 32 CFR 219.102(f).This sample set comprised of Chikungunya virus positive and negative samples, as determined by real-time PCR [28]. Total nucleic acid was extracted from each sample using the Qiagen EZ1 XL Advanced with the EZ1 Virus Mini kit 2.0 and tested using the BPDA.

## Results

### Preliminary assay evaluation

We developed a Broad Pathogen Detection Assay (BPDA) for use with the NanoString nCounter platform in order to quickly screen a sample for multiple pathogens in a single tube reaction. This assay consisted of 195 detection probes targeting 164 different viral, bacterial, and parasitic pathogens of concern for human health. Initial testing using the highest concentration of organism available showed positive detection of multiple pathogens including EBOV, MARV, *P*. *falciparum*, and *C*. *burnetii* (see Table 1 for a selected list and the S1 File for a full detection list). However, some pathogens such as Crimean-Congo hemorrhagic fever virus (CCHFV) were not detected even at this high concentration (Table 1). A multiplexed target enrichment (MTE) step utilizing a complex PCR to amplify the pathogen-specific probe hybridization site was used for use prior to detection with the BPDA to mitigate this issue. Incorporating this upfront target enrichment step increased the assay sensitivity as shown by the now positive detection of CCHFV and increased read counts for almost all pathogens tested (Table 1).

**Table 1:** Selected pathogen detection with and without MTE.

Pathogen	BPDA			MTE- BPDA		
	Concentration (PFU/ml) ^1^	Counts	P/N	Concentration (PFU/ml) ^1^	Counts	P/N
CHIKV	3.36 x 10^7^	129,978/129,908	+/+	3.35 x 10^5^	237,479/293,522	+/+
*Coxiella burnetti*^2^	1.66 x 10^10^	4,746/4,695	+/+	1.66 x 10^10^	200,996/221,019	+/+
		3,611/3,550	+/+		169,451/178,909	+/+
		5,898/5,911	+/+		318,421/330,036	+/+
CCHFV	1.28 x 10^7^	9/19	-/-	1.28 x 10^7^	3,680/479,843	+/+
DENV-1	2.18 x 10^5^	31,379/35,025	+/+	2.18 x 10^3^	579,678/495,661	+/+
DENV-2	2.74 x 10^6^	85,904/80,761	+/+	2.74 x 10^4^	848,967/805,148	+/+
DENV-3	1.03 x 10^5^	89,696/77,774	+/+	1.03 x 10^4^	6,257/4,653	+/+
DENV-4	7.30 x 10^5^	127,598/124,171	+/+	7.30 x 10^3^	809,068/984,413	+/+
EBOV (Kikwit)	1.82 x 10^6^	489/444	+/+	2.28 x 10^6^	365,650/475,458	+/+
Lassa virus (Josiah)	9.58 x 10^4^	1,457/1,268	+/+	9.58 x 10^4^	555,878/495,615	+/+
MARV (Musoke)	9.12 x 10^7^	2,021/1,880	+/+	9.13 x 10^7^	624,376/850,269	+/+
*P*. *falciparum*^2^	1.51 x 10^5^	144/167	+/+	1.51 x 10^5^	13,044/4,510	+/+
		27/25	+/+		88,1234/830,759	+/+
*P*. *vivax*	UND	44/27	+/+	UND	69,440/54,202	+/+
		70/91	+/+		255,078/327,349	+/+
		90/76	+/+		384,695/433,329	+/+
Yellow fever virus	2.26 x 10^6^	15,863/16,485	+/+	2.26 x 10^5^	569,402/544,624	+/+
Zika virus	2.62 x 10^6^	129,112/132,539	+/+	2.62 x 10^4^	258,748/337,182	+/+

UND = concentrations not determinable

^1^*C*. *burnetii*, *P*. *falciparum*, and *P*. *vivax* are listed as genome equivalents/ml

^2^Three probes were included for *C*. *burnetii* and two probes for *P*. *falciparum*

### MTE target enrichment

**Fig 1:**
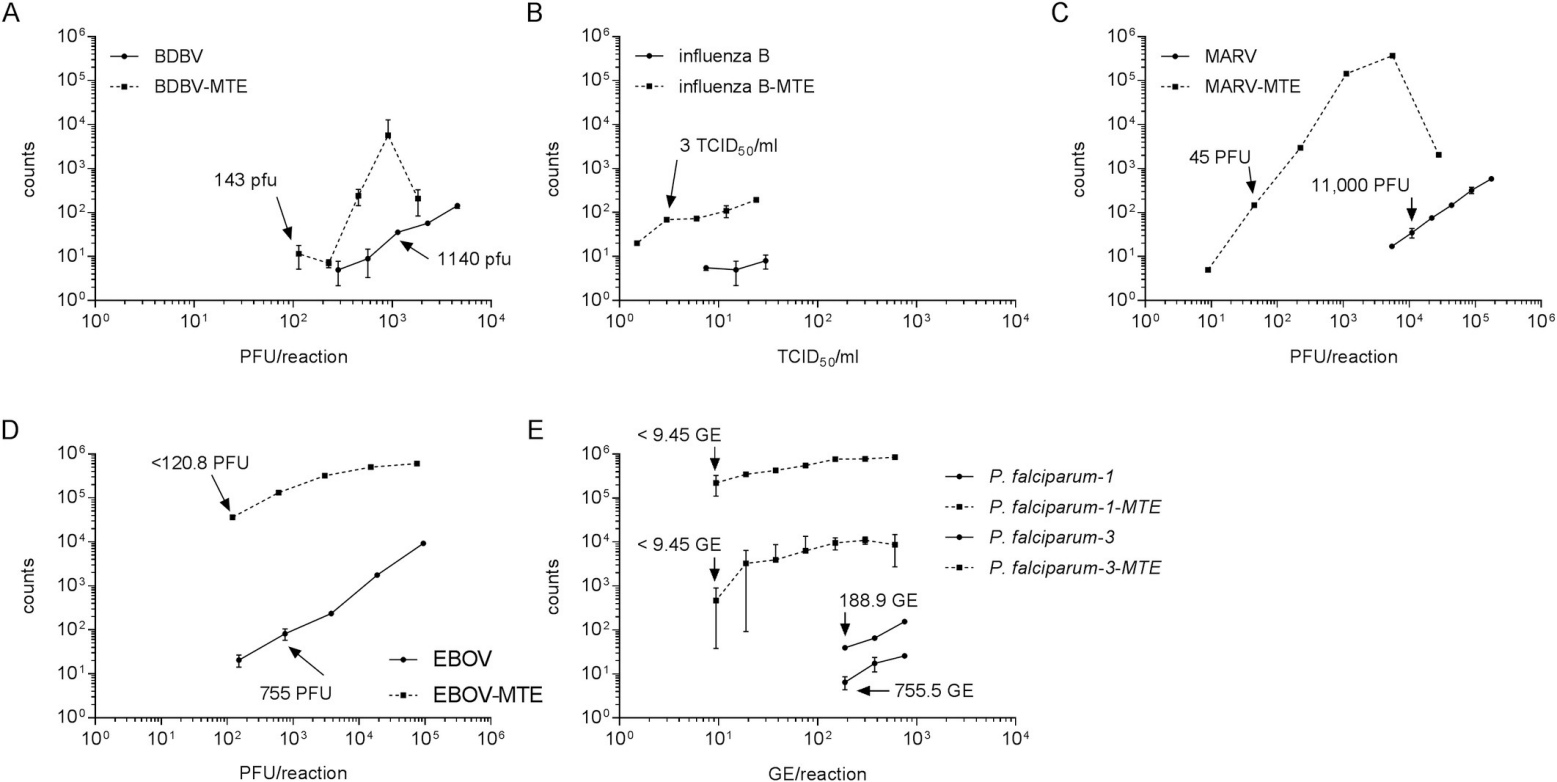
Improved PCR detection following MTE. BDBV (A), influenza B strain 38 (B), MARV (C), EBOV (D), and *P*. *falciparum* (E) nucleic acid were serially diluted. One dilution series was amplified by MTE prior to being run in the SYBR Green assay. The amount of pathogen-specific nucleic acid was determined by real-time PCR using primers internal to the MTE amplification primers. Fig B is shown as a dilution factor because influenza B strain 38 did not titer. Samples were run in triplicate, and the error bars represent the standard deviation of the mean. A two-way ANOVA with Bonferroni determined all dilutions for all viruses tested were significantly different with the exception of the highest influenza B virus concentration (~10^3^ PFU/rxn).

Having shown the effectiveness of the MTE step for increasing assay sensitivity, we wanted to further assess the target enrichment capability of this method by comparing the amount of target amplicon present with and without MTE. Comparing real-time PCR results for the MTE amplified and non-amplified reactions, MTE enrichment showed a decrease in Cq values, indicating an increase in the target amplicon (Fig 1). Statistical analysis (two-way ANOVA with Bonferroni correction) identified that all points for each virus were significantly different with the exception of the highest influenza B virus concentration.

### Analytical performance of the BPDA

**Fig 2:**
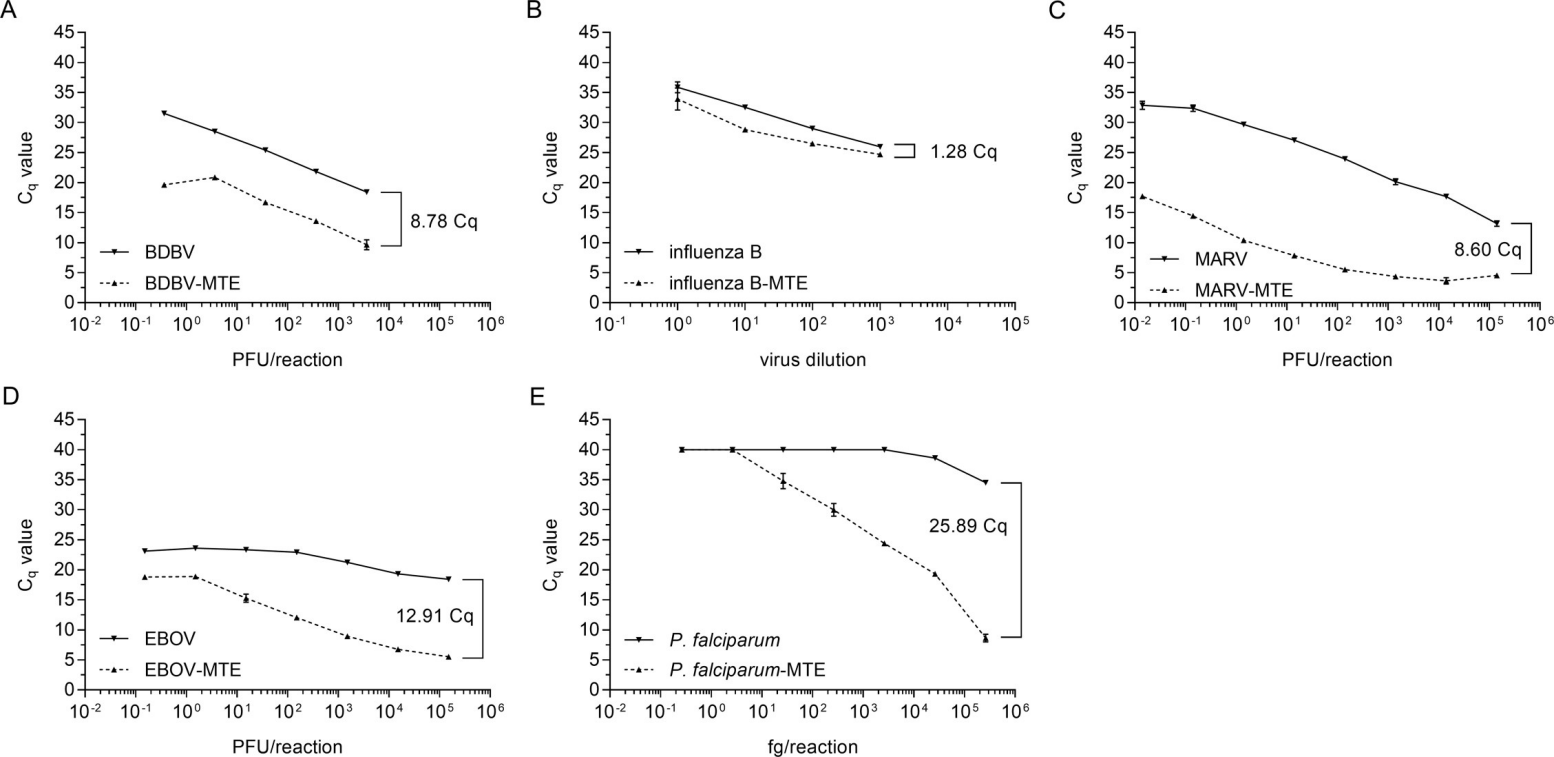
Preliminary LOD determination by the NanoString broad pathogen detection panel. BDBV (A), influenza B virus (B), MARV (C), EBOV (D), and *P*. *falciparum* (E) nucleic acid were serially diluted and assayed with and without MTE in triplicate. Arrows indicate the lowest concentration where all three replicates were positive (the preliminary LOD). Error bars show the range of the two samples. Influenza B virus was undetectable without amplification.

Assay limit of detection (LOD) studies were conducted with serially diluted organism in order to define assay performance for medical relevant concentrations of the tested organism. Incorporating the MTE enrichment improved detection and lowered LODs (Fig 2). This improvement was most notable for influenza B virus which was undetectable without enrichment but tested positive following MTE (Fig 2B). Similarly, the preliminary LOD for MARV improved from 2.2 x 10^6^ PFU/ml without MTE to 3.75 x 10^4^ PFU/ml after enrichment (Fig 2D). Generally, the assays were highly specific for the targeted organism. For example, BPDA showed positive results for only *P*. *falciparum* while other *Plasmodium* species including *knowlesi*, *malariae*, *ovale*, and *vivax* were called as true negatives.

To confirm assay reproducibility at the preliminary LOD, ten replicates of BDBV, influenza B virus, and MARV were tested with and without MTE (Table 2). For influenza B virus, no replicates tested positive at the highest concentration used; however, MTE incorporation resulted in repeated detection of all viruses. In addition, comparison of BPDA performance to real-time PCR, the current gold standard for molecular based pathogen detection, showed real-time RT-PCR was the more sensitive technique (Table 2).

**Table 2:** Confirmation of LOD.

Pathogen	BPDA			MTE- BPDA		
	Concentration (PFU/ml)	Detection	Ave ± STDEV	Concentration (PFU/ml)	Detection	Ave ± STDEV
Influenza B virus	ND	ND	ND	3 TCID_50_/ml	10/10	19.9±7.67
BDBV	2.28 x 10^5^	10/10	132.3±22.7	3.58 x 10^4^	8/10	427.6±292.6
MARV (Angola)	2.2 x 10^6^	10/10	40.3±7.72	3.75 x 10^4^	9/10	25,810±8,645

ND = not detectable at this concentration

### Clinical sample testing

Testing EBOV and DENV-3 spiked into whole blood at three different concentrations showed the clinical applicability of this assay. Application of the MTE did not impact the overall testing results, but MTE increased the number of EBOV-specific counts for all dilutions and replicates (Table 3). DENV-3 tested positive with all replicates and dilutions; however, MTE did not result in increased DENV-3 counts (Table 3). Interestingly, the pre-amplification of DENV-3 signatures resulted in a lower number of counts as compared to the same samples without MTE, potentially suggesting suboptimal amplification for that DENV-3 isolate.

**Table 3:** Mock clinical detection.

Pathogen	Concentration (PFU/ml)	BPDA		MTE- BPDA	
		Detection	Average ± STDEV	Detection	Average ± STDEV
EBOV	6.8 x 10^6^	3/3	1,637 ± 172	3/3	328,220 ± 10,142
	8.5 x 10^3^	3/3	410 ± 19	3/3	124,547 ± 16,564
	2.13 x 10^3^	3/3	84 ± 14	3/3	36,752 ± 7,590
DENV-3	2.06 x 10^4^	3/3	593 ± 16	3/3	62 ± 8
	4.11 x 10^3^	3/3	6,644 ± 1,312	3/3	1,616 ± 848
	820	3/3	1,151 ± 181	3/3	173 ± 10

Further characterization of the clinical utility of the BPDA showed positive detection across 14 de-identified, human clinical samples with potential Chikungunya virus (CHIKV) infections (Table 4). Both the BPDA and the MTE-BPDA assays correctly identified the 12 real-time RT-PCR positive samples and the two negative samples (Table 4). Incorporating the MTE component increased the number of CHIKV counts by 1–2 log for all of the positive samples tested.

**Table 4:** Clinical patient sample detection for CHIKV.

sample	BPDA		MTE- BPDA		CHIKV qRT-PCR
	detection	raw counts	detection	raw counts	
sample 1	0/2	0/0	0/2	0/0	-
sample 2	2/2	3,996/3,554	2/2	713,689/932,015	24.56
sample 3	2/2	4,919/5,289	2/2	497,152/804,985	24.8
sample 4	0/2	0/0	0/2	0/0	-
sample 5	2/2	9,393/7,363	2/2	878,959/819,611	22.89
sample 6	2/2	4,563/4,037	2/2	910,763/825,149	24.61
sample 7	1/1	4,136	1/1	385,505	22.6
sample 8	1/1	31,122	1/1	192,935	19.37
sample 9	1/1	14,859	1/1	297,920	19.71
sample 10	1/1	34,271	1/1	530,413	19.72
sample 11	1/1	21,292	1/1	515,250	21.13
sample 12	1/1	24,289	1/1	634,621	20.72
sample 13	1/1	102,701	1/1	216,161	16.59
sample 14	1/1	30,855	1/1	391,354	20.22

## Discussion

Positively identifying the etiologic agent for an acute febrile illness can be critical for ensuring appropriate administration of treatment and supportive care; however, proper identification can be challenging. Highlighting the importance of broad pathogen screening and appropriately fielded diagnostics, a recent study by Schoepp and colleagues found ~70% of the suspected Lassa fever patients admitted to the Lassa Fever Ward in Kenema, Sierra Leone, were negative for both Lassa virus and the malaria parasite, both hyperendemic pathogens in the region [29]. This study found serological evidence of filovirus infection (EBOV and MARV) in the years prior to the explosive Ebola virus disease outbreak in West Africa [29], and it is likely that previous infections with EBOV as well as MARV were misidentified as severe malaria or Lassa fever. Accurate testing of these acute febrile patients with multiplexed assays could have identified the risk of an EBOV outbreak earlier.

Here, we developed and evaluated a highly multiplexed, broad pathogen detection assay for use following negative detection using singleplex assays (ex. real-time PCR). This assay targets 164 different human pathogens of public health concern and includes viruses, bacteria, and parasites. We included multiple organisms with overlapping clinical presentation, such as *Plasmodium*, Lassa virus, and Ebola virus. We also included a large number of less common organisms in order to maximize the diagnostic potential of the assay. While the assay performed well without target enrichment, applying MTE prior to running on the NanoString platform greatly improved assay sensitivity. While extensive primer optimization was not conducted in this study, such an optimization would likely improve the overall assay sensitivity and the detection variation we observed. As we were unable to do this optimization, all testing was conducted with and without MTE. Overall, 98 of the 164 pathogens on the panel that we had available for testing were positively identified including endemic pathogens to West Africa such as EBOV, Lassa virus, dengue virus, and the malaria parasite *P*. *falciparum*. Assay run time, from start to finish, is approximately 27 hours with approximately one hour (or 30 minutes without the MTE) of hands-on time. Future efforts include the acquisition and testing of the remaining pathogens on the panel.

Characterization of the BPDA using mock clinical and clinical samples showed the efficacy of this assay for detecting pathogen in patient samples. Specifically, testing of spiked human serum showed positive detection of EBOV and DENV-3 at clinically relevant concentrations. In addition to mock clinical samples, the assays correctly identified all CHIKV human clinical samples, demonstrating the ability to correctly identify pathogens from natural infections. These studies were in agreement with real-time RT-PCR testing establishing preliminary 2-by-2 testing that would be required for regulatory use.

There are a variety of easy to use multiplexed assays described in the literature [6, 7, 30, 31]; however, there are inherent limitations in multiplexability within a single assay. Other technologies offer higher levels of multiplexing through microarray [32, 33] and next-generation sequencing [16–19, 34]. However, these assays are highly technical, and the large number of targets makes full validation of each signature highly challenging in both cost and time. Furthermore, clinical validation of these assays would be further complicated by the regulatory requirement to validate each potential organism the assay could detect [35, 36]. Preliminary limit of detection (5 dilutions in triplicate for 164 organisms) and confirmation of the preliminary limit of detection (twenty replicates of 164 organisms) testing alone would require 2,460 reactions at approximately $100 USD/reaction (~$575,000 USD). Mock clinical testing would further expand the testing numbers and cost.

Ideally, comparison of primer/primer interactions and a direct comparison of each target to a gold standard (ex. real-time PCR) would be performed for diagnostic applications. Within current regulatory paradigm, this type of validation also remains cost prohibitive similar to other highly multiplexed assays (ex. microarray and next-generation sequencing assays) as this would require independent testing of each target on the panel in a statistically robust manner. However, results presented here provide proof of concept testing results and the framework for such a validation by demonstrating the proof of concept utilization of this technology for infectious disease diagnostics.

## Supporting information

S1 File The Supporting Information File includes tables containing: 1) all organisms and strains tested and the BPDA test results; 2) primers utilized in the MTE reaction; and 3) the nested real-time PCR primers. (XLSX)
